# Usefulness of Intraoperative Neurophysiological Monitoring During the Clipping of Unruptured Intracranial Aneurysm: Diagnostic Efficacy and Detailed Protocol

**DOI:** 10.3389/fsurg.2021.631053

**Published:** 2021-02-26

**Authors:** Dougho Park, Byung Hee Kim, Sang-Eok Lee, Eunhwan Jeong, Kwansang Cho, Ji Kang Park, Yeon-Ju Choi, Suntak Jin, Daeyoung Hong, Mun-Chul Kim

**Affiliations:** ^1^Department of Rehabilitation Medicine, Pohang Stroke and Spine Hospital, Pohang-si, South Korea; ^2^Department of Neurology, Pohang Stroke and Spine Hospital, Pohang-si, South Korea; ^3^Department of Anesthesiology, Pohang Stroke and Spine Hospital, Pohang-si, South Korea; ^4^Department of Radiology, Pohang Stroke and Spine Hospital, Pohang-si, South Korea; ^5^Department of Neurosurgery, Pohang Stroke and Spine Hospital, Pohang-si, South Korea

**Keywords:** intracranial aneurysms, postoperative complication, intraoperative neurophysiological monitoring, motor evoked potential, somatosensory evoked potential

## Abstract

**Background:** Intraoperative neurophysiological monitoring (IONM) has been widely applied in brain vascular surgeries to reduce postoperative neurologic deficit (PND). This study aimed to investigate the effect of IONM during clipping of unruptured intracranial aneurysms (UIAs).

**Methods:** Between January 2013 and August 2020, we enrolled 193 patients with 202 UIAs in the N group (clipping without IONM) and 319 patients with 343 UIAs in the M group (clipping with IONM). Patients in the M group were intraoperatively monitored for motor evoked potentials (MEPs) and somatosensory evoked potentials (SSEPs). Irreversible evoked potential (EP) change was defined as EP deterioration that did not recover until surgery completion. Sustained PND was defined as neurological symptoms lasting for more than one postoperative month.

**Results:** Ten (3.1%) and 13 (6.7%) in the M and N groups, respectively, presented with PND. Compared with the N group, the M group had significantly lower occurrence rates of sustained PND [odds ratio (OR) = 0.36, 95% confidence interval (CI) = 0.13–0.98, *p* = 0.04], ischemic complications (OR = 0.39, 95% CI = 0.15–0.98, *p* = 0.04), and radiologic complications (OR = 0.40, 95% CI = 0.19–0.82, *p* = 0.01). Temporary clipping was an independent risk factor for ischemic complications (ICs) in the total patient group (OR = 6.18, 95% CI = 1.75–21.83, *p* = 0.005), but not in the M group (OR = 5.53, 95% CI = 0.76–41.92, *p* = 0.09). Regarding PND prediction, considering any EP changes (MEP and/or SSEP) showed the best diagnostic efficiency with a sensitivity of 0.900, specificity of 0.940, positive predictive value of 0.321, negative predictive value (NPV) of 0.997, and a negative likelihood ratio (LR) of 0.11.

**Conclusion:** IONM application during UIA clipping can reduce PND and radiological complications. The diagnostic effectiveness of IONM, specifically the NPV and LR negative values, was optimal upon consideration of changes in any EP modality.

## Introduction

Intracranial aneurysms occur in 3–5% of adults; moreover, upon rupture, it causes subarachnoid hemorrhage (SAH), which results in critical neurological damage or, in the worst case, death (1, 2). Studies have reported an annual rupture risk of 0.5–1.4% for unruptured intracranial aneurysm (UIA), with size, morphology, age, and previous UIA rupture being identified as risk factors (2–4). Microsurgical UIA clipping and endovascular coil embolization are two procedures widely used for UIA repair and rupture prevention (5). In some cases, microsurgical clipping is essential to preventing UIA rupture; however, caution should be exercised as surgery bears risks of systemic complications, including pneumonia, seizure, infection, cerebral ischemia, and intracranial hemorrhage (6, 7). As the surgery is quite sensitive in nature, the process should be performed precisely. Specifically, blood flow into the aneurysm should be completely blocked, with maximum patient safety being simultaneously guaranteed (8).

Currently, intraoperative neurophysiological monitoring (IONM) is widely employed during UIA clipping. Recent studies have reported that IONM during UIA clipping could reduce postoperative complications, especially ischemic complications (ICs), using appropriate rescue interventions (9, 10). Studies on UIA clipping conducted before 2010 that did not use IONM reported an IC rate of 6–14% (6, 7, 11). Contrastingly, recent studies using IONM have reported IC rates of 1–8%, which emphasizes the effectiveness of IONM (10, 12).

This study aimed to describe our experience with IONM during UIA clipping. Specifically, we aimed to assess the postoperative outcomes according to our IONM protocol, as well as the risk factors for postoperative IC. Moreover, we aimed to perform an in-depth review of intraoperative evoked potential (EP) changes and the occurrence of postoperative neurologic deficits (PNDs).

## Materials and Methods

### Patient Inclusion and Assessments

We enrolled patients who underwent microsurgical UIA clipping at our hospital between January 2013 and August 2020. They were divided into two groups based on IONM usage. Patients who underwent UIA clipping before March 2017 (January 2013–February 2017) were enrolled in the non-IONM usage group (N group). On the other hand, patients who underwent UIA clipping between March 2017 and August 2020 were included in the IONM usage group (M group). The exclusion criteria were as follows: (1) presence of ruptured intracranial aneurysms; (2) administration of simultaneous treatment procedures other than clipping (e.g., cerebral bypass surgery and endovascular coiling); (3) presence of other intracranial pathologies, including infection, tumor, or vascular malformation; (4) unobtainable EP owing to underlying disease; (5) severe neurological disorders due to underlying disease [modified Rankin scale (mRS) score ≥2); and (6) absence of 1-month postoperative follow-up.

All patients underwent brain computed tomography (CT) scans within 24 postoperative hours. Additional brain CT or magnetic resonance imaging (MRI) scans were performed at the discretion of a neurosurgeon and radiologist based on clinical symptoms or postoperative CT scan findings.

Regarding clinical assessments, the mRS score was obtained before surgery, as well as at 24 h and 1 month after surgery. PND was defined as a postoperative increase in the mRS score compared with the preoperative mRS score. Transient and sustained PNDs were defined as the presentation of postoperative neurological symptoms that recovered and did not recover, respectively, within one postoperative month. IC was defined as having PND caused by cerebral infarction.

### IONM Protocol and Related Surgical Procedures

IONM was performed using the XLTEK Protektor 32 (Natus Medical, Oakville, Canada). Motor evoked potentials (MEPs), somatosensory evoked potentials (SSEPs), and electroencephalograms were used as IONM modalities during UIA clipping. EPs were bilaterally measured in both the upper and lower limbs of participants.

For MEP stimulation, transcranial electrical stimuli were delivered through subdermal needle electrodes. The stimulus intensity was set to 200–350 V; moreover, stimuli were delivered using five pulse trains with a pulse duration of 0.5 ms at an inter-stimulus interval of 1–4 ms. The filter range was set to 10–3,000 Hz. The electrodes were placed at C1 and C2 following the International 10–20 system. For MEP recording, subcutaneous needle electrodes were placed at the flexor carpi radialis and abductor pollicis brevis muscles in the upper extremities, as well as at the tibialis anterior and abductor hallucis (AH) muscles in the lower extremities. SSEP stimuli were delivered using square-wave 0.3-ms electrical pulses at a frequency of 1.75 Hz. The stimulus intensity was set to 25 and 30 mA for the median and tibial SSEP, respectively. For SSEP recording, the electrodes were placed at C3′, C4′, Cz, and just above the fifth cervical spinous process. The reference electrode was placed at Fpz. The filter range was set at 30–1,000 Hz.

**Figure 1:**
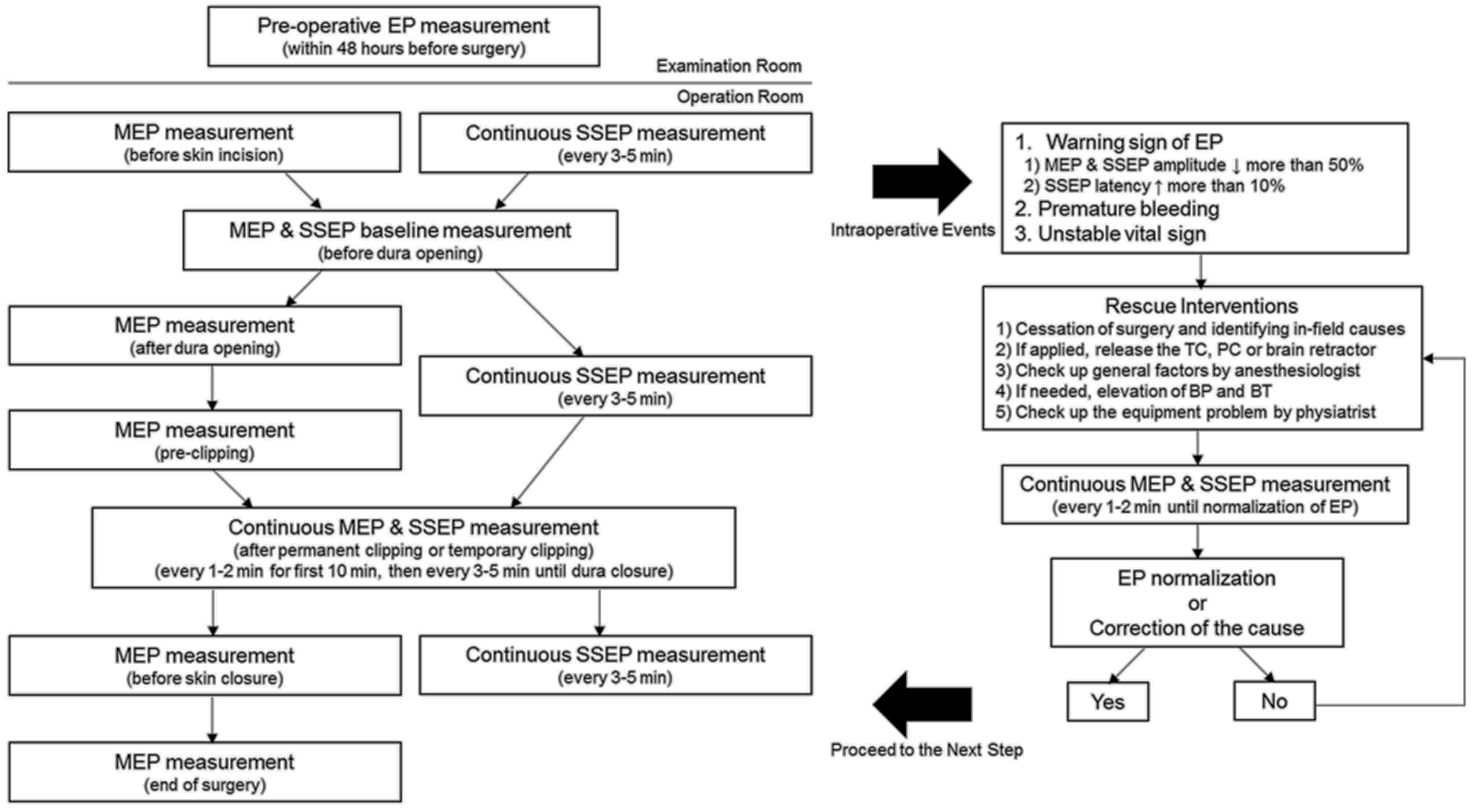
Intraoperative neurophysiological monitoring protocol for unruptured intracranial aneurysm clipping. MEP was performed after anesthetic induction, just before dura opening, after dura opening, before clipping, after clipping, after dura closure, and after skin closure. SSEP was continuously checked every 3–5 min. The baseline EP was obtained just before the dura opening. Regular MEP checkup was conducted in case of TC, PC, or intraoperative events, such as the presence of SSEP warning sign, premature bleeding, and unstable vital sign. Preoperative EP examinations were performed within 48 preoperative hours for excluding possible mechanical or technical errors in the operation room. *MEP*, motor evoked potential; *SSEP*, somatosensory evoked potential; *EP*, evoked potential; *TC*, temporary clipping; *PC*, permanent clipping; *BP*, blood pressure; *BT*, body temperature.

Figure 1 presents our hospital's IONM protocol during UIA clipping. Routine MEP examinations were performed after anesthetic induction, just before dura opening, after dura opening, before clipping, after clipping, after dura closure, and after skin closure. After permanent clipping (PC), continuous MEP monitoring was performed every 1–2 min for the first 10 min. Subsequently, we checked the MEP at 3–5-min intervals until dura closure. Furthermore, we conducted unscheduled continuous MEP monitoring every 1–2 min under the following circumstances: (1) when temporary clipping (TC) was applied; (2) in the case of an intraoperative event, including premature bleeding or unstable vital signs; or (3) a warning sign of other EP. Regarding SSEP, continuous checkup was intraoperatively performed every 3–5 min. Similar to MEP, we conducted continuous monitoring every 1–2 min during the aforementioned surgical events.

The baseline EP was obtained just before the dura opening. The warning criteria were defined as follows: (1) a >50% decrease in MEP amplitude, (2) a >50% decrease in SSEP amplitude, and (3) a delay of >10% in SSEP latency (13, 14). In case there was a change in EP meeting the warning criteria, the surgeon stopped the surgery and clarified the cause in the surgical field. In case a TC was applied, the clip was immediately released. In the case of PC application, the clip was first released; subsequently, the PC was reapplied after the EP normalized. For cases other than those aforementioned, we checked whether the retractor being intraoperatively used was applying pressure on the brain and whether there were changes in blood pressure (BP), body temperature (BT), or anesthetic state. In cases without accidental bleeding, the anesthesiologist increased the BP and took the necessary measures for maintaining optimal BT. The physiatrist checked mechanical errors and wiring connection defects while performing continuous monitoring, every 1–2 min. All patients in the M group underwent preoperative EP examinations within 48 preoperative hours to exclude possible mechanical or technical errors in the operation room.

Reversible EP change was defined as EP deterioration that intraoperatively recovered. Contrastingly, we defined irreversible EP change as EP deterioration that did not recover until the end of surgery. Moreover, to identify the IONM predictive value, the EP change patterns were classified into four categories: MEP alone, SSEP alone, all EP changes (both MEP and SSEP), and any EP changes (MEP and/or SSEP).

TC was implemented for aneurysm remodeling before PC, when it was necessary to reduce intra-aneurysmal pressure according to its size and shape. In case of premature aneurysmal bleeding, TC was also implemented. In most cases, only proximal TC was applied; however, both proximal and distal TCs were applied in the event of premature aneurysmal bleeding. In the former cases, normotension was maintained after TC, but in the latter cases, systolic BP was maintained at around 150 mmHg with phenylephrine infusion. The maximum duration of a single TC was 10 min; the duration should not exceed that. After removal of the TC, surgical procedures were stopped for the time the TC was applied, so that the distal blood flow was sufficiently compensated. If vasoconstriction was confirmed, papaverine was sprayed into the surgical field.

After the application of the PC, all patients were observed for 10 min without any additional surgical procedure. During that period, systolic BP was maintained at around 150 mmHg, while the physiatrist continuously checked for changes in EP. Further, all patients underwent microvascular Doppler ultrasonography (DU) and indocyanine green (ICG) angiography to confirm incomplete clipping or presence of blood flow disturbance in the parent artery.

### Anesthesia

Total intravenous anesthesia (TIVA) was applied to all patients in the M group. TIVA was induced using propofol (3–5 mg/ml) and remifentanil (3–5 ng/ml). TIVA was maintained through continuous infusion of propofol (2.5–3.5 mg/ml) and remifentanil (2.5–4.5 mg/ml). Here, we used a DPS Orchestra (Fresenius Kabi, Frankfurt, Germany) infusion pump, and anesthetic levels were maintained using a bispectral index ranging from 30 to 60. A neuromuscular blocking agent was used as a single bolus (rocuronium bromide, 0.4–0.5 mg/kg) before intubation. No inhalation agent was used.

### Statistical Analysis

Continuous variables were expressed as mean ± SD or median (range). Categorical variables were expressed as frequencies and proportions. An independent *t*-test was used for between-group comparisons of continuous variables. Moreover, between-group comparisons of categorical variables and postoperative outcomes were performed using the chi-square test and Fisher's exact test. A multivariate logistic regression model was used to identify the risk factors for postoperative IC. The Mann–Whitney test was used to compare the intraoperative reaction time and deterioration duration between MEP and SSEP. Statistical analyses were conducted using SPSS 22.0 (IBM, Armonk, NY, USA). To calculate sensitivity, specificity, positive predictive value (PPV), negative predictive value (NPV), and negative likelihood ratio (LR), we used GraphPad Prism 9 (GraphPad Software, San Diego, CA, USA).

## Results

### Baseline Characteristics

This study enrolled 319 patients with 343 UIAs in the M group and 193 patients with 202 UIAs in the N group. In both groups, there were fewer men than women (27.0 and 26.9%, respectively). The mean age of those in the M group (61.27 ± 9.04 years) was significantly higher than that in the N group (59.02 ± 10.28 years, *p* = 0.01). The operation time in the M group (223 ± 65.83 min) was significantly shorter than that in the N group (267 ± 75.48 min, *p* < 0.001). There was no significant between-group difference in the vascular risk factors, TC application frequencies, multiple UIA operation frequencies, and adjacent perforator frequencies. Moreover, there was a between-group difference in the operation side, but not in the vessel location and UIA size (Table 1).

**Table 1:** Baseline characteristics of patients.

**Variable**		**M group**	**N group**	***p*-value**
Patients, *n*		319	193	
Age (years)		61.27 ± 9.04	59.02 ± 10.28	0.01
Sex, male (%)		86 (27.0)	52 (26.9)	0.99
Vascular risk factors, *n* (%)	Hypertension	169 (53.0)	97 (50.3)	0.55
	Diabetes	51 (16.0)	22 (11.4)	0.15
	Dyslipidemia	77 (24.1)	33 (17.1)	0.06
	Heart problems^a^	21 (6.6)	17 (8.8)	0.35
	Previous CVI	17 (5.3)	11 (5.7)	0.86
	Smoking	46 (14.4)	34 (17.6)	0.33
Operation duration (min)		223 ± 65.83	267 ± 75.48	<0.001
Temporary clip, *n* (%)		26 (8.2)	13 (6.7)	0.56
Multiple UIA operation, *n* (%)		24 (7.5)	8 (4.7)	0.13
Aneurysm, *n*		343	202	
Side, *n* (%)				0.006
	Right	184 (53.6)	80 (39.6)	
	Left	116 (33.8)	87 (43.1)	
	Central	43 (12.5)	35 (17.3)	
Vessel, *n* (%)				0.13
	MCAB	175 (51.0)	97 (48.0)	
	MCA	46 (13.4)	22 (10.9)	
	Acom	43 (12.5)	35 (17.3)	
	ACA	30 (8.7)	11 (5.4)	
	Pcom	19 (5.5)	17 (8.4)	
	Acho	28 (8.2)	15 (7.4)	
	ICA	2 (0.6)	5 (2.5)	
Size (mm)		3.96 ± 1.49	3.80 ± 1.45	0.21
Adjacent perforator, *n* (%)		56 (16.3)	33 (16.3)	>0.99

*CVI, cerebrovascular infarction; UIA, unruptured intracranial aneurysm; MCAB, middle cerebral artery bifurcation; MCA, middle cerebral artery; Acom, anterior communicating artery; ACA, anterior cerebral artery; Pcom, posterior communicating artery; Acho, anterior choroidal artery; ICA, internal carotid artery; M group, monitored; N group, non-monitored*.

a *Coronary artery diseases or symptomatic arrhythmias*.

### Overall Outcomes

In the N group, PND developed in 13 (6.7%) patients; among them, three (1.6%) and 10 (5.2%) were transient and sustained, respectively. There were nine, one, one, and two cases of cerebral ischemia, SAH, intracranial hemorrhage (ICH), and ischemia with falx hemorrhage, respectively. Radiologic complications occurred in 17 (8.8%) patients, with four patients showing asymptomatic radiological positives [two falx hemorrhages, one subdural hematoma (SDH), and one ICH].

**Figure 2:**
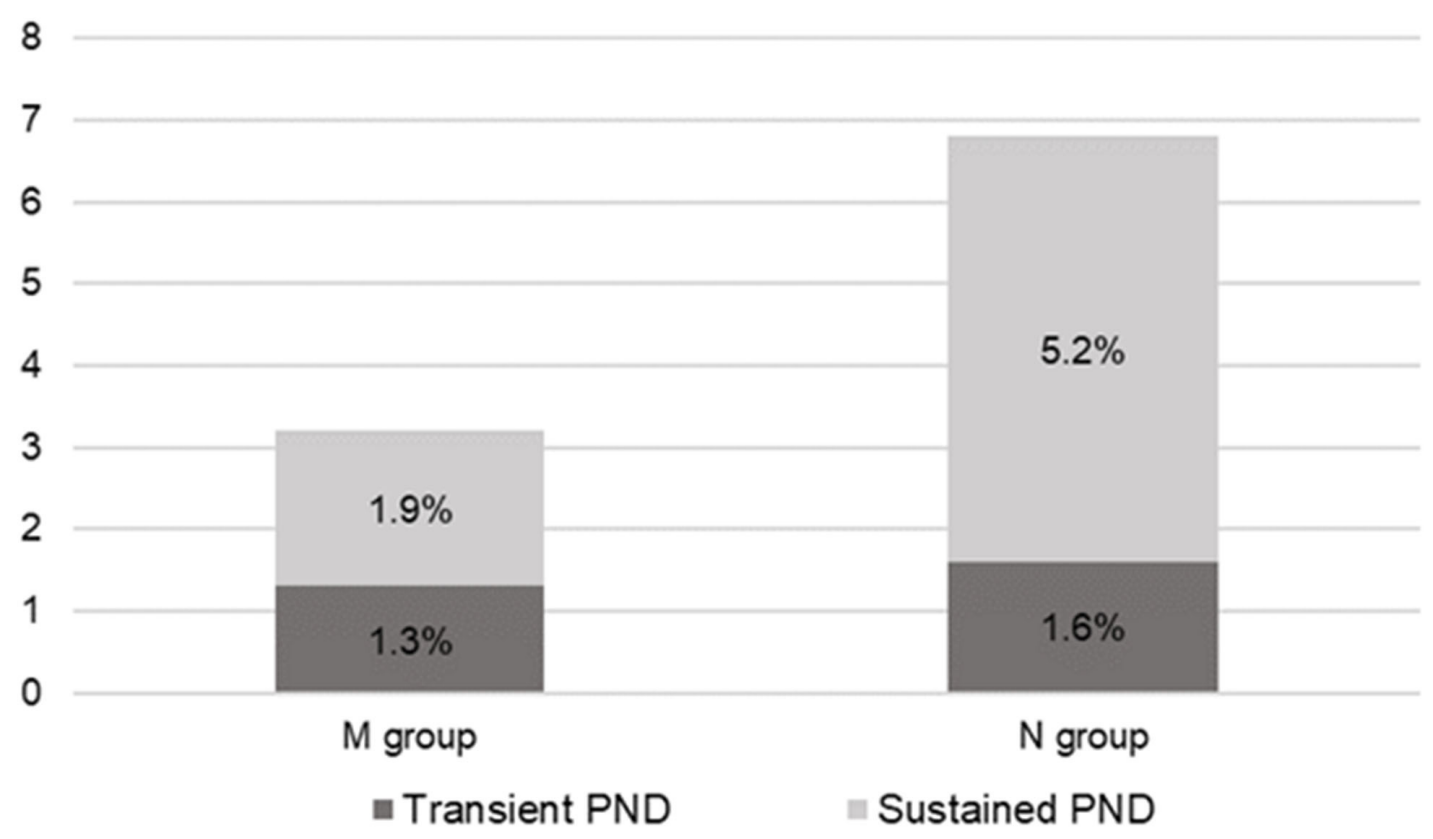
Overall postoperative neurologic deficit (PND) rate. In the M group, four (1.3%) patients presented transient PND and six (1.9%) patients presented sustained PND. Meanwhile, in the N group, three (1.6%) patients presented transient PND and 10 (5.2%) patients presented sustained PND. *M* group, monitored; *N* group, non-monitored.

In the M group, 10 (3.1%) patients had PND; among them, four (1.3%) and six (1.9%) were transient and sustained, respectively (Figure 2). Further, there were seven, two, and one case(s) of cerebral ischemia, SDH, and SAH, respectively. Radiologic complications occurred in 12 (3.8%) patients. Three patients had asymptomatic radiological positives (one minimal frontal ICH, one SDH, and one frontal low density). One patient presented with radiologically negative transient weakness.

**Figure 3:**
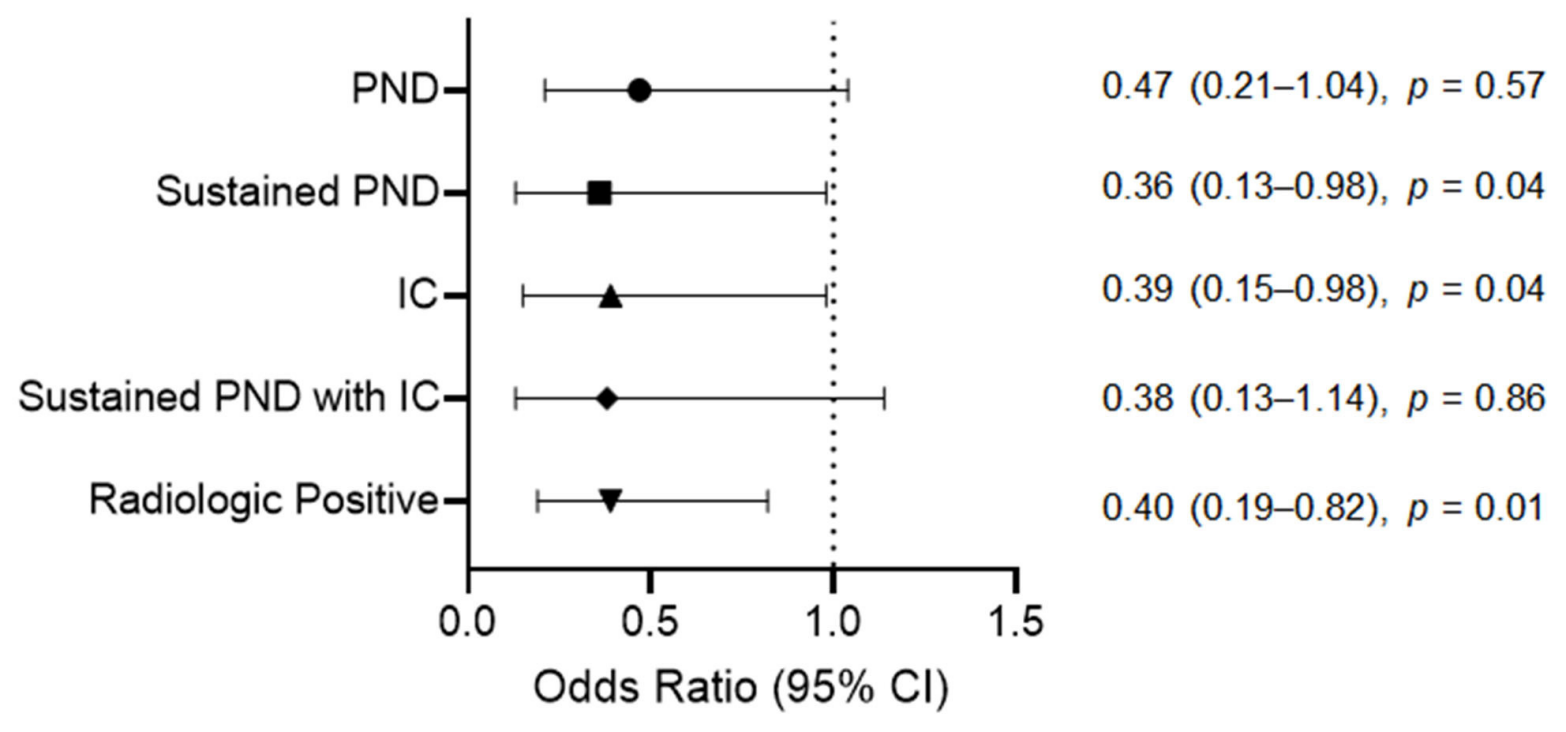
Odds ratios for the postoperative complications after unruptured intracranial aneurysm clipping according to the intraoperative neurophysiological monitoring implementation. For sustained PND, IC, and radiologic positive, the M group had a significantly lower incidence rate than the N group. The incidence rate of overall PND and sustained PND due to IC between the M and N groups showed no significant difference. *PND*, postoperative neurologic deficit; *IC*, ischemic complication; *CI*, confidence interval; *M* group, monitored; *N* group, non-monitored.

There was no significant between-group difference in the overall PND incidence [odds ratio (OR) = 0.47, 95% confidence interval (CI) = 0.21–1.04, *p* = 0.57]. We categorized postoperative complications into subgroups for detailed analyses. The M group had significantly fewer sustained PND cases (6, 1.9%) than the N group (10, 5.2%) (OR = 0.36, 95% CI = 0.13–0.98, *p* = 0.04). The IC incidence rate was significantly lower in the M group (7, 2.2%) than in the N group (11, 5.7%) (OR = 0.39, 95% CI = 0.15–0.98, *p* = 0.04). Regarding sustained PND with IC, the M group had five (1.6%) cases and the N group had eight (4.1%) cases, with no significant between-group difference (OR = 0.38, 95% CI = 0.13–1.14, *p* = 0.86). The incidence rate of radiologic positives was significantly lower in the M group than in the N group (OR = 0.40, 95% CI = 0.19–0.82, *p* = 0.01) (Figure 3).

### Risk Factors for Ischemic Complications

We performed a risk factor analysis for postoperative IC. Multivariate analysis using the total patient group revealed that TC application significantly increased the IC incidence (OR = 6.18, 95% CI = 1.75–21.83, *p* = 0.005). However, multivariate analysis using the M group did not reveal significance of TC application as a risk factor for IC (OR = 5.53, 95% CI = 0.76–41.92, *p* = 0.09). The other variables considered in the multivariate analysis, including IONM, age, sex, operation time, multiple UIA surgeries, vascular risk factors, aneurysm size, vessel territory, and adjacent perforator, were not significant risk factors for IC (Table 2). There was no significant association between the vessel type where the UIA was located and IC in the total patient group, M group, and N group (*p* = 0.24, *p* = 0.89, and *p* = 0.63, respectively). Among the IC cases, there was also no significant difference in aneurysm location between the M group and the N group (*p* = 0.26) (Supplementary Table 1).

**Table 2:** Risk factor analysis for ischemic complications after UIA clipping.

**Variable**	**Total patients (*****n*** **= 512)**		**Patients with IONM (*****n*** **= 319)**	
	**OR (95% CI)**	***p*-value**	**OR (95% CI)**	***p*-value**
M group (with IONM)	0.52 (0.17–1.55)	0.24		
Age (per year)	1.04 (0.98–1.10)	0.25	1.11 (0.98–1.25)	0.10
Sex (female)	0.45 (0.14–1.49)	0.19	0.60 (0.08–4.74)	0.63
OP duration (per 30 min)	1.20 (0.98–1.47)	0.07	1.14 (0.82–1.60)	0.44
Multiple UIA OP	0.68 (0.71–6.55)	0.74	2.47 (0.20–30.83)	0.48
Hypertension	0.62 (0.22–1.76)	0.37	0.88 (0.16–4.69)	0.88
Diabetes	0.68 (0.14–3.43)	0.64	0.98 (0.14–7.00)	0.99
Dyslipidemia	0.94 (0.25–3.58)	0.93	0.64 (0.06–6.64)	0.71
Heart problems^a^	2.22 (0.49–10.02)	0.30	5.43 (0.67–44.28)	0.11
Previous CVI	1.81 (0.34–9.78)	0.49	1.81 (0.14–24.17)	0.65
Smoking	3.24 (1.00–10.46)	0.05	2.46 (0.26–23.36)	0.43
Temporary clipping	6.18 (1.75–21.83)	0.005^b^	5.53 (0.76–41.92)	0.09
UIA size (per mm)^c^	1.21 (0.91–1.58)	0.18	1.19 (0.67–2.13)	0.56
Non-MCA territory^c^	1.47 (0.45–4.82)	0.52	1.57 (0.13–19.25)	0.72
Adjacent perforator^c^	1.18 (0.30–4.66)	0.81	1.64 (0.11–24.72)	0.72

*IONM, intraoperative neurophysiological monitoring; OP, operation; UIA, unruptured intracranial aneurysm; CVI, cerebrovascular infarction; MCA, middle cerebral artery; OR, odds ratio; CI, confidence interval; M group, monitored*.

a *Coronary artery diseases or symptomatic arrhythmias*.

b *Statistically significant by multivariate analysis*.

c *Multivariate analysis excluding multiple UIA cases*.

### Intraoperative EP Changes and Postoperative Neurologic Deficit

In the M group, 28 (8.8%) patients presented any intraoperative EP changes meeting the warning criteria; among them, 19 (6.0%) patients revealed reversible EP changes, with one patient showing PND. Irreversible EP changes were observed in nine (2.8%) patients; among them, eight presented PND. Twelve (3.8%) patients showed MEP changes; among them, four had irreversible MEP changes and PND. Of the eight patients with reversible MEP changes, one presented PND. Twenty-one (6.6%) patients showed changes in SSEP; among them, six had irreversible SSEP changes, with five showing PND. Among the 15 patients with reversible SSEP changes, only one patient presented PND. There were five (1.6%) patients showing both EP changes simultaneously; among them, one had irreversible changes in both MEP and SSEP while one had irreversible and reversible SSEP and MEP changes, respectively. Both cases presented with accompanying PND. All three patients with reversible changes in both MEP and SSEP lacked PND (Supplementary Table 2).

Reversible EP changes were confirmed for eight MEPs and 15 SSEPs, which showed a deterioration duration of 22.5 min (17–64 min) and 16 min (7–50 min), respectively. There was no significant difference between both EPs (*p* = 0.30). Regarding the response time of each EP after a given intraoperative event, it took 8 min (4–14 min) and 15 min (2–38 min) for 14 MEP and 21 SSEP changes, respectively. MEP responded significantly faster than SSEP (*p* = 0.02). The following events caused intraoperative EP changes: PC for 16 patients (57.1%), TC for eight patients (28.6%), dura opening for two patients (7.1%), cortical bleeding for one patient, and traction injury for one patient.

Among the 10 patients with PND in the M group, nine presented with any EP changes, with eight presenting more than one irreversible EP change. One patient had false-negative findings. Additionally, among the 24 (7.5%) patients who underwent multiple UIA surgeries in the M group, two (8.3%) patients showed EP changes, with one of them having PND.

Table 3 summarizes the characteristics, IONM findings, radiologic findings, and functional assessments of patients with PND in the M group.

**Table 3:** Patients with postoperative neurologic deficits in the M group.

		**Aneurysm**			**IONM findings**		**Radiologic findings**	**Functional assessments**		
**Case** **no**.	**Age** **/sex**	**Location**	**Size** **(mm)**	**TC (min)**	**MEP**	**SSEP**	**CT and** **/or MRI**	**mRS 1 day**	**mRS 1 month**	**Symptom** **descriptions**
18	63/M	Left MCAB	4.2	No	Amp↓ (IR)	No change	Negative	2	0	Leg weakness, dizziness
101	71/F	Acom	2.5	No	Loss (IR)	No change	Right frontal SDH	1	1	Leg weakness
111	54/F	Right Pcom	6.5	7	No change	No change	Right BG and thalamic infarction	3	2	Hemiparesis
167	81/M	Left MCAB	5.4	No	Amp↓ (IR)	No change	Left frontal infarction with SDH	1	3	Hemiparesis
179	70/F	Left ICA	3.5	No	Amp↓ (IR)	Amp↓ (IR)	SAH	1	0	Leg weakness
233	67/F	Left MCAB and MCA	5 & 4	No	No change	Amp↓ (IR)	Left MCA borderzone infarction	2	1	Hemiparesis
236	60/M	Left MCAB	3.9	No	No change	Amp↓ (R)	Left MCA infarction	1	1	Dysarthria, hemiparesis
261	75/F	Acom	2	No	No change	Amp↓ (IR)	Left ACA infarction	3	3	Hemiparesis, motor aphasia
266	80/F	Left MCAB	5.2	4.5	No change	Amp↓ (IR)	Left temporal cortex infarction	2	0	Dysarthria
278	51/M	Left MCAB	2.9	no	Amp↓ (R)	Amp↓ (IR)	Left MCA infarction	1	0	Dizziness, gait disturbance

*M group, monitored; IONM, intraoperative neurophysiological monitoring; TC, temporary clipping; MEP, motor evoked potential; SSEP, somatosensory evoked potential; CT, computed tomography; MRI, magnetic resonance imaging; mRS, modified Rankin scale; MCAB, middle cerebral artery bifurcation; Acom, anterior communicating artery; Pcom, posterior communicating artery; ICA, internal carotid artery; MCA, middle cerebral artery; IR, irreversible deterioration; R, reversible deterioration; SDH, subdural hemorrhage; BG, basal ganglia; SAH, subarachnoid hemorrhage; ACA, anterior cerebral artery*.

### Diagnostic Efficacy and Predictive Value of IONM

Based on the aforementioned results, we calculated the diagnostic efficacy and predictive value of IONM during UIA clipping. Table 4 summarizes the sensitivity, specificity, PPV, NPV, and LR negative for IONM according to each category of EP change pattern.

**Table 4:** Sensitivity, specificity, PPV, and NPV of multimodal evoked potential monitoring during UIA clipping.

**Applied modalities**	**Sensitivity** **(95% CI)**	**Specificity** **(95% CI)**	**PPV (95% CI)**	**NPV** **(95% CI)**	**Negative** **LR**
Changes in any EP (MEP or/and SSEP)	0.900 (0.596–0.995)	0.939 (0.906–0.960)	0.321 (0.179–0.507)	0.997 (0.981–1.000)	0.11
Changes in all EP (both MEP and SSEP)	0.200 (0.036–0.510)	0.990 (0.972–0.997)	0.400 (0.071–0.769)	0.975 (0.951–0.987)	0.81
Changes in MEP	0.500 (0.237–0.763)	0.977 (0.954–0.989)	0.417 (0.193–0.681)	0.984 (0.962–0.993)	0.51
Changes in SSEP	0.600 (0.313–0.832)	0.952 (0.922–0.971)	0.286 (0.138–0.500)	0.987 (0.966–0.995)	0.42
Reversible EP changes	0.100 (0.005–0.404)	0.941 (0.909–0.963)	0.053 (0.002–0.246)	0.970 (0.944–0.984)	0.96
Irreversible EP changes	0.800 (0.490–0.965)	0.997 (0.982–1.000)	0.889 (0.565–0.994)	0.994 (0.977–0.999)	0.20

*CI, confidence interval; PPV, positive predictive value; NPV, negative predictive value; LR, likelihood ratio; EP, evoked potential; MEP, motor evoked potential; SSEP, somatosensory evoked potential*.

Generally, IONM showed high specificity and NPV, but relatively low sensitivity and PPV. Any EP change showed the highest sensitivity of 0.9, highest NPV of 0.997, and lowest LR negative value of 0.11. For all EP changes considered, there was high specificity and low sensitivity of 0.99 and 0.2, respectively, with the negative LR being the worst. The use of either MEP or SSEP as the sole modality had a low sensitivity of 0.5 and 0.6, respectively; moreover, the corresponding negative LR values were relatively high (0.51 and 0.42, respectively).

We conducted further analysis regarding the recovery of EP changes. For irreversible EP changes, the specificity was 0.997, which was the highest among all analyses, while the NPV and negative LR were 0.994 and 0.2, respectively.

### Temporary Clipping

TC was applied to 26 (8.2%) patients in the M group; among them, eight (30.8%) presented EP change. Two patients developed IC; among them, one patient presented with an irreversible SSEP change after 4.5 min of TC application. In the 1-month follow-up, however, the patient had recovered to normal functional level. The other patient underwent TC for 7 min and was postoperatively suspected of right basal ganglia infarction and sustained PND. However, IONM revealed false-negative findings. In the N group, TC was applied to 13 (6.7%) patients; among them, three patients presented IC, with each patient having TC applied for 4, 5, and 7 min, respectively. Moreover, they all presented with postoperative basal ganglia infarction.

In the M group, 7.7% of the patients presented with IC following TC application. This percentage was numerically, but not significantly, lower than that of the N group (23.1%) (OR = 0.28, 95% CI = 0.05–1.58, *p* = 0.31). There was no significant between-group difference in the time taken to apply TC [M group, 5 min (1–10 min); N group, 5 min (4–7 min); *p* = 0.99]. In the M group, seven (24.1%) and 22 (75.9%) TCs were applied for premature bleeding control and aneurysm remodeling, respectively. Meanwhile, in the N group, six (46.2%) and seven (53.9%) TCs were applied for premature bleeding control and aneurysm remodeling, respectively. The M and N groups showed the highest TC application frequency in middle cerebral artery bifurcation (MCAB; 55.2 and 53.8%, respectively). The aneurysm location in TC cases and the proportion of the TC application purpose did not significantly differ between the M and N groups (*p* = 0.91 and *p* = 0.17, respectively) (Supplementary Table 3).

### Noteworthy Cases

**Figure 4:**
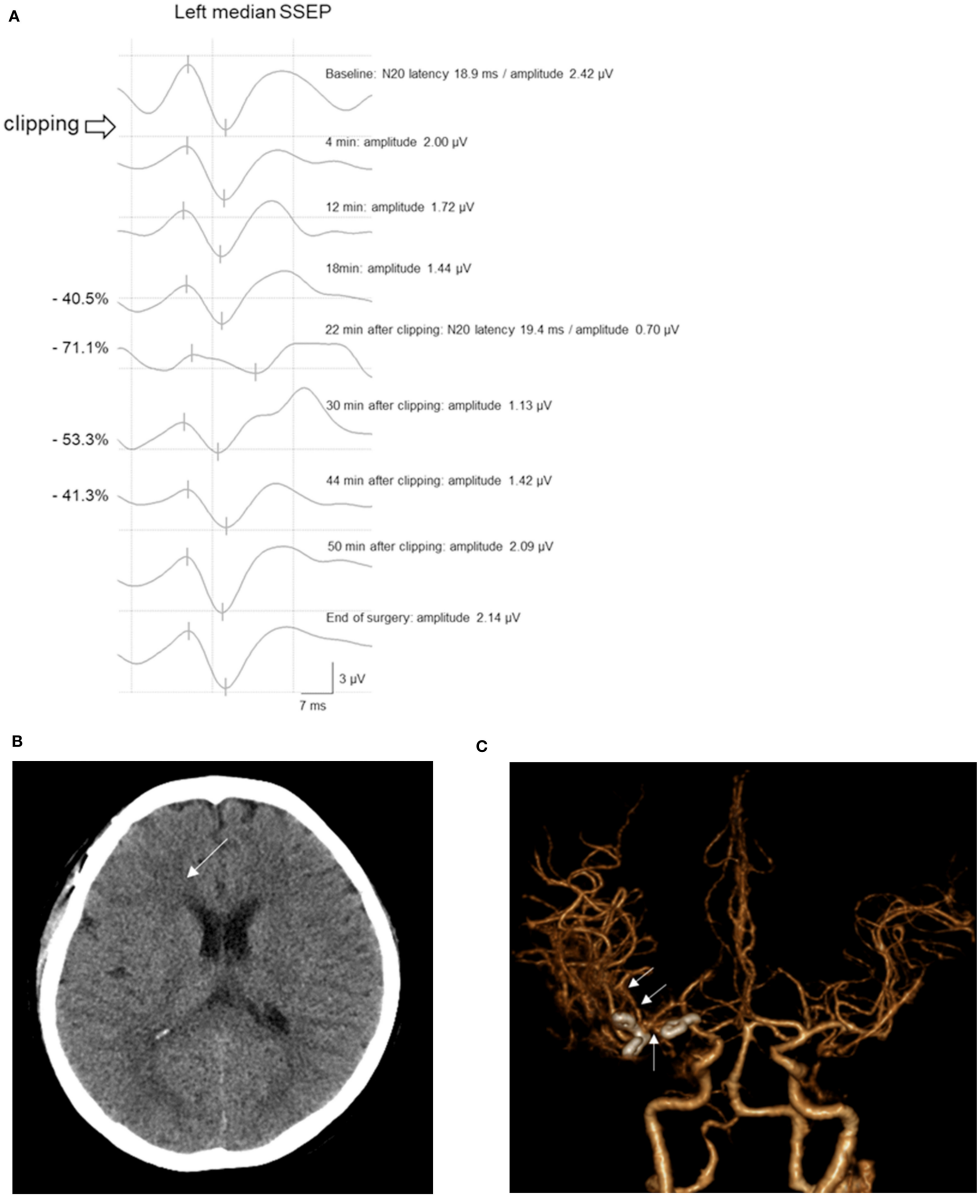
A 55-year-old woman with MCAB and ICAB UIA underwent microsurgical clipping. **(A)** After permanent clipping of the ICAB, the amplitude of the left median SSEP decreased by >50%. After 22 min, the left median SSEP amplitude recovered and maintained in the acceptable range until the end of surgery. **(B)** The postoperative brain CT scan showed a slightly low density in the right frontal area (*arrow*). **(C)** CT angiography revealed vasospasms of the right M1 and M2 (*arrows*) with clipping of the MCAB and ICAB UIA. Inconsistent with the imaging findings, the patient did not present neurological symptoms. *MCAB*, middle cerebral artery bifurcation; *ICAB*, internal carotid artery bifurcation; *UIA*, unruptured intracranial aneurysm; *SSEP*, somatosensory evoked potential; *CT*, computed tomography.

Case 193 underwent surgical repair of a right MCAB and internal carotid artery bifurcation UIA. The amplitude of the left median SSEP decreased to 71.1% within 22 min after PC placement. Subsequently, the clip was immediately released and the cause was checked. BP and BT were elevated according to the protocol. Approximately 20 min after the clip release, the left median SSEP amplitude recovered to 41.3% lower than the baseline value, which was within the acceptable range. Then, the PC was reapplied. Further, the SSEP amplitude did not reach the warning criteria range. Subsequently, the SSEP amplitude further increased and recovered to baseline levels. Postoperative CT scans revealed a slightly low density in the right frontal area, as well as M1 and M2 vasospasms (Figure 4). However, the patient did not show any postoperative neurological symptoms and was neurologically normal within a 1-month follow-up period.

**Figure 5:**
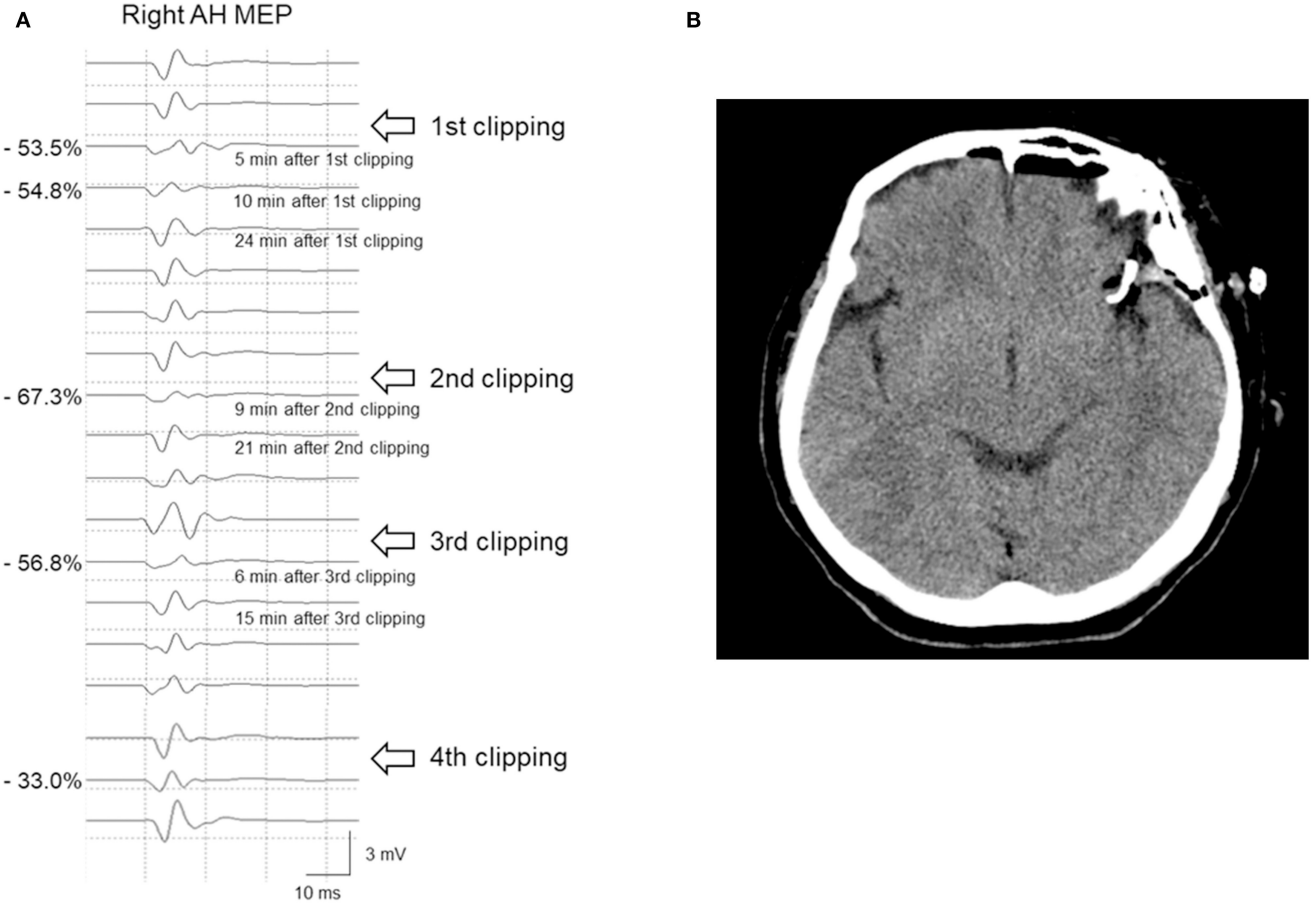
A 65-year-old woman with left MCAB aneurysm who underwent microsurgical clipping. **(A)** After permanent clipping, the MEP amplitude of the right AH muscle decreased by >50%. Repeated clip repositioning was performed due to MEP warning signs. Finally, after the fourth repositioning of the main clip, there was no further reduction in MEP amplitude. **(B)** The postoperative brain CT scan showed no abnormal findings, and she lacked neurological sequelae. *MCAB*, middle cerebral artery bifurcation; *MEP*, motor evoked potential; *AH*, abductor hallucis; *CT*, computed tomography.

Case 8 underwent clipping of a left MCAB UIA. Five minutes after PC placement, the right AH MEP amplitude decreased by 53.5%. Consequently, as per our hospital's protocol, the PC was immediately released. Moreover, BP and BT were increased, with subsequent progress being observed. The patient's MEP subsequently recovered and the PC was reapplied. However, after 9 min, the AH MEP amplitude decreased by 67.3%, which prompted the release of the PC and the surgeon to examine other causes, with none being identified. After >10 min, the MEP amplitude recovered. Six minutes after the third clipping attempt, the AH MEP amplitude decreased by 56.8%. The operating surgeon considered that the middle cerebral artery (MCA) would go into traction during MCAB clipping, which would affect the blood flow. Therefore, the surgeon changed directions and repositioned the fourth clip, with the MEP remaining stable. During the four aforementioned events, SSEP did not show any change meeting the warning criteria; further, neither ICG angiography nor DU checkup revealed abnormal findings. The patient's postoperative CT findings were unremarkable and there were no neurological symptoms (Figure 5).

**Figure 6:**
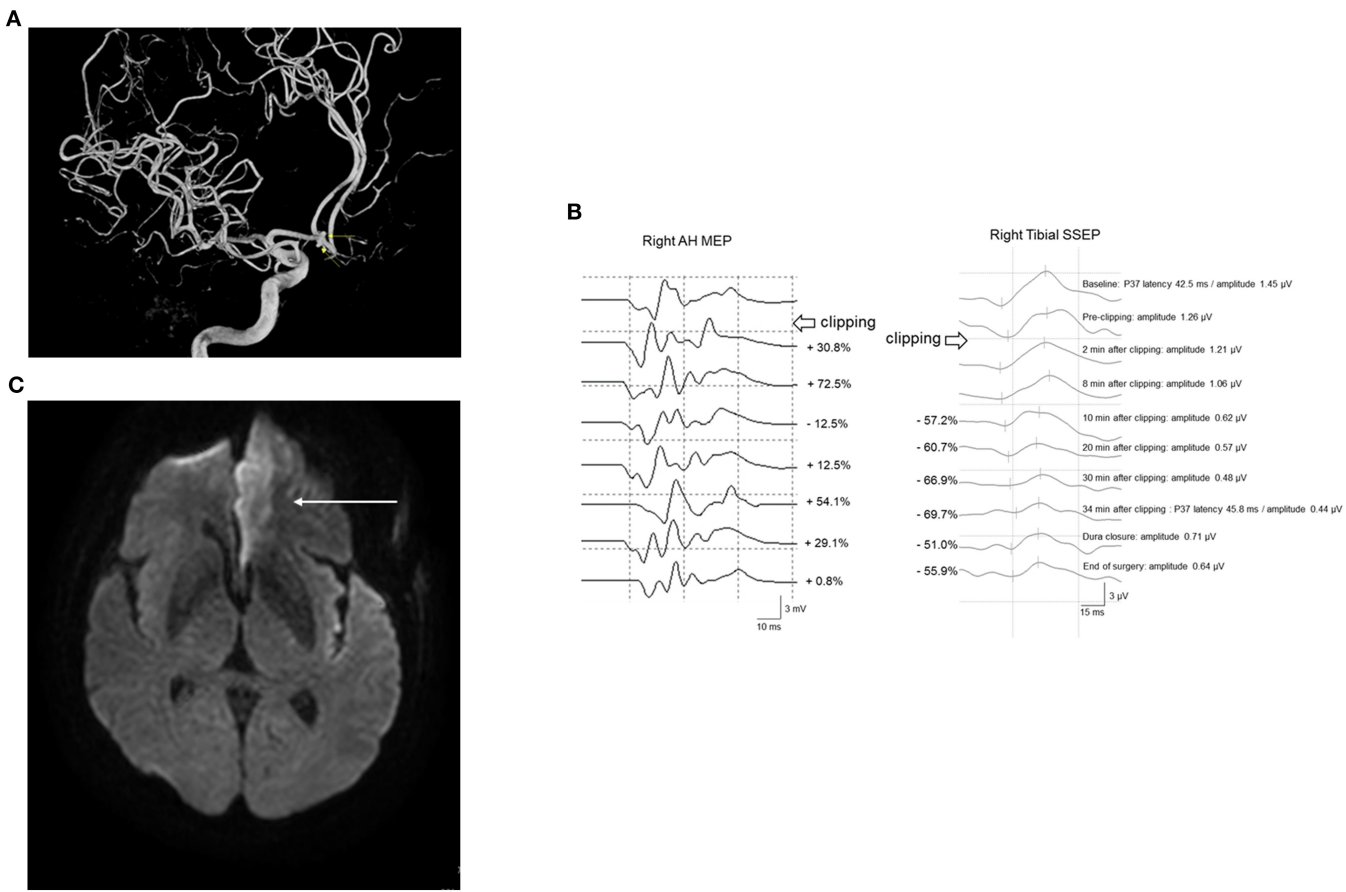
A 61-year-old woman with Acom UIA who underwent microsurgical clipping. **(A)** Angiographic reconstruction imaging revealed double Acom UIA (*arrows*). **(B)** After permanent clipping, the amplitude of the right tibial SSEP decreased by >50%, while the corresponding right AH MEP remained within an acceptable range. A postoperative brain CT scan showed no definite change. **(C)** However, subsequent diffusion-weighted brain imaging revealed acute left ACA infarction (*arrow*). She postoperatively presented with right leg weakness and mild motor aphasia. *Acom*, anterior communicating artery; *UIA*, unruptured intracranial aneurysm; *SSEP*, somatosensory evoked potential; *AH*, abductor hallucis; *MEP*, motor evoked potential; *CT*, computed tomography; *ACA*, anterior cerebral artery.

Case 261 underwent UIA clipping of the anterior communicating artery (Acom). The patient's right tibial SSEP amplitude decreased by 57.2% 10 min after PC. The operation was stopped, followed by rescue interventions. However, the amplitude of the right tibial SSEP remained below 50% of the baseline. Simultaneous MEP monitoring of the right lower extremity showed no significant change. As the SSEP amplitude continuously deteriorated, PC reapplication was attempted after confirming that MEP remained within an acceptable range. Subsequently, ICG angiography and DU findings were examined, with the MEP being continuously monitored until the end of surgery. The amplitude of SSEP reduced by >50% until the end of the operation. Postoperative diffusion MRI revealed left anterior cerebral artery (ACA) territory infarction; moreover, the patient complained of right leg weakness and motor aphasia (Figure 6).

## Discussion

In this study, the PND incidence in the M group was more than twice lower than that in the N group. Both the IC and sustained PND incidence, but not the overall PND, showed a significant between-group difference. There have been several studies on the effectiveness of IONM during UIA clipping surgeries. Greve et al. (15) reported a numerically, but not significantly, lower PND occurrence rate in the MEP- and SSEP-monitored group than that in the control group (5.8 and 7.3%, respectively). Byoun et al. (16) reported a significant decline in the IC rates after MCA UIA clippings in the SSEP-monitored group compared with the non-SSEP-monitored group (0.9 *vs*. 5.6%). Moreover, Yue et al. (17) reported no significant difference in the motor improvement rate between the monitored and non-monitored groups at the discharge time. However, at the last follow-up, the monitored group showed a significantly higher motor improvement rate. However, this is a cohort study, which includes cases of ruptured aneurysms. Since the study differs in baseline characteristics from ours, the scope for direct comparison is limited. Nasi et al. (10) conducted a meta-analysis of the aforementioned studies and found a significantly lower risk of overall PND occurrence in the IONM group. However, regarding sustained PND, the IONM group showed a non-significant trend of lower risk. Overall, each study partially differed in their results. We speculated that the discrepancies arose because the EP modalities considered were slightly different. Further, there were differences in the PND definition, and the set duration for considering sustained PND also differed.

In our study, compared with the N group, the M group showed a significantly low incidence rate of radiologic positives. This could be attributed to the relatively high proportion of asymptomatic radiological positives in the N group. This indicates that IONM usage contributes to a reduced incidence of postoperative asymptomatic hemorrhage or infarction. Additionally, even with the occurrence of radiologic complications in the M group, the symptoms could be minimized or prevented through aggressive intraoperative rescue interventions, which was confirmed by case 193. In this case, MCA vasospasm occurred after PC placement, which was revealed by postoperative CT angiography. However, since PC was applied while continuously increasing the BP and monitoring SSEP improvement, PND was prevented even with radiologic infarction.

IONM not only reduces PND but also influences the decision regarding whether to perform surgery, as well as intraoperative decisions. In our study, patients in the N and M groups were enrolled for 50 and 42 months, respectively. However, the numbers of patients in the N and M groups were 193 and 319, respectively, even though those in the M group were significantly older than those in the N group. This suggests that IONM usage could be a favorable factor for the surgical treatment of UIA. In our study, the M group showed a significantly shorter operation time than the N group. Given that 8.8% of our patients showed intraoperative changes in EP, additional time could have been required during such cases to allow rescue interventions for handling EP changes. Nevertheless, IONM could have provided safety clues in the negative cases, which allowed faster operation (18). There have been limited reports regarding the duration of UIA clipping surgeries. In 2016, a study conducted on 115 patients who underwent anterior choroidal artery UIA clipping with IONM reported a surgery duration of 240.79 ± 90.60 min (19). This duration was slightly longer than that of our M group and shorter than that of our N group. However, this interpretation should be made with extra caution. Specifically, it is difficult to attribute the reduced operation times solely to IONM. Given that the N group was enrolled much earlier than the M group, the differences in operation time could have resulted from differences in the skill levels of some surgeons or surgical procedures. Specifically, surgeons might have improved their skills over time.

Furthermore, IONM could affect TC application. TC is crucially involved in improving the clipping integrity and reducing the risk of premature intraoperative aneurysm rupture (20, 21). In our study, there was no significant between-group difference in the frequency of TC application. Meanwhile, the proportion of TC for aneurysm remodeling was higher in the M group than in the N group. This might mean that IONM allowed surgeons to apply TC more actively, even when there was no statistical difference between groups. No standard duration for TC application has been established. The reported duration of TC application has ranged from 3 to 23 min depending on the target vessel features; moreover, intermittent TC has been shown to reduce the infarction rate (22–24). In our study, six patients in the M group underwent intermittent TC application. Applying intermittent TC could take up to 12 min in one patient. We found that IONM usage allowed flexibility in setting the time for TC application rather than having to adhere to specifically set times. Our risk factor analysis revealed that only TC was an independent risk factor for IC, which is consistent with previous findings (25). However, TC was not a significant risk factor in the analysis of the M group alone. The IC incidence rates after TC application in the M and N groups were 7.7 and 23.1%, respectively, which suggests that IONM could lower the IC risk when applying TC. However, there was no significant between-group difference in the incidence rates, which could be attributed to the small sample size that underwent TC. Thus, we inferred that IONM could not only lower the IC rate but also provide safety clues to surgeons for TC application for UIA (20).

We found that the median response times of MEP and SSEP to intraoperative events were 8 and 12 min, respectively. There have been few studies on the response times of MEP and SSEP, which could be attributed to the difficulty in determining the exact time and cause of the intraoperative event. Moreover, regarding MEP, continuous monitoring could not be performed in numerous cases during the non-critical portion of surgery. Therefore, it is difficult to consider response times as exact and reliable. Dengler et al. (26) performed bypass surgeries with IONM for repairing giant aneurysms and reported an average time for MEP change after TC of 144 ± 52 s, which significantly differed from the 8 min observed in our M group. However, this period was analyzed using only five patients after inducing complete main artery occlusion. Consequently, there was a significant difference between the previous study and ours. We observed TC-induced EP change in only 28.6% of the overall EP changes. In most cases, the response time was slower than the previously reported value since there was no direct occlusion of the parent artery flow. Staarmann et al. (23) separately reported the response times of MEP and SSEP. Calculation of the median value of response times based on their report revealed that the response times of MEP and SSEP were 5 min (3.5–21 min) and 3 min (1.5–17 min), respectively. Compared with our findings, Staarmann et al. reported relatively shorter response times, especially those for SSEP. Since they reported that 12 out of 15 SSEP changes occurred after TC, there could have been a difference in response time since the TC rate was much lower in our patient group. In case the main flow was not restricted, the EP response might not have been immediate. Even in TC cases involving total parent vessel occlusion, it took 2–5 min to recognize MEP changes (24, 26). Specifically, the surgeon should not exclude the possibility of clip release or repositioning even without changes immediately after clipping. Similarly, the physiatrist should maintain a steady EP checkup for a certain period after clipping. According to our protocol, for TC and PC applications, continuous monitoring should be maintained until dura closure.

In our IONM protocol, the EP data obtained immediately before dura opening were used as baseline data. In particular, for MEP, determining the time to obtain baseline data is important. This is because the MEP data obtained immediately after anesthesia may have low effectiveness as baseline data given the effect of the single bolus of a neuromuscular blocking agent during intubation. For this reason, previous studies have used baseline MEP data obtained just before dura opening (27, 28). Another study used EP data after opening the dura (before clipping) as baseline data (29). However, according to our experience, EP could change given the impact of cerebrospinal fluid drainage or brain shrinkage after opening the dura. Additionally, after dura opening, parenchymal compression due to retractor application might occur during the dissection procedure, which could affect the EP findings. Therefore, we considered the best time for obtaining the baseline EP data as just before dura opening.

When establishing the IONM protocol during UIA clipping, we aimed to maximize the monitoring capacity with minimum disturbance of the surgeon. Unfortunately, few studies on UIA clipping with IONM have reported the timing and frequency of intraoperative MEP and SSEP stimulation. Moreover, a standardized protocol remains to be established. Some studies have briefly mentioned this topic. For example, Choi et al. (27) reported designation of different times depending on the intraoperative circumstances; further, they conducted MEP and SSEP stimulations before skin incision, duration incision, and vessel manipulation. Moreover, they conducted MEP stimulations after retractor application, arachnoid membrane dissection, TC, PC, and unexpected event occurrence. Staarmann et al. (23) described the following protocol where they conducted intraoperative EP monitoring every 5–10 min at non-critical surgical portions. After the application of either TC or PC, they performed continuous EP monitoring every 1–2 min for 30 min. Li et al. (28) only described intraoperative MEP monitoring every 3–5 min. Based on previous reports, we designated several routine checkup times for the MEP. Meanwhile, for SSEP, continuous checkup was performed during the entire surgery. SSEP can be regularly monitored regardless of the surgical procedure; however, during MEP stimulation, the surgical procedure should be momentarily paused for safety. Therefore, given multidisciplinary agreement, we attempted to establish a protocol that sensitively responds to surgical events, is efficient, and does not burden surgeons. Based on our experience, we established our current IOMN protocol presented in Figure 1. The detailed description of our IONM protocol could be a reference material for using IONM during UIA clipping surgeries, as well as contribute to the standardization of an IONM protocol in the future.

Consistent with previous studies, analysis of the diagnostic accuracy of IONM revealed a relatively low sensitivity and PPV as well as a high specificity and NPV (9, 30, 31). It appears that rescue interventions during IONM-positive cases lower the proportion of true positives. Defining false positives in IONM is very complicated; moreover, it is virtually impossible to accurately distinguish between a true positive reversed through rescue interventions and a pure false positive (12). In case 8, repeated PC repositioning was attempted due to a recurring decrease in MEP amplitude. Eventually, surgery was completed after confirming MEP improvement, with no PND occurring. In the presence of MCA vessel traction, surgery completion with only checking of the flow through ICG angiography and DU could have increased the likelihood of perforator infarction. However, aggressive rescue intervention allowed PND prevention. Although PND did not occur, this case could not be classified as a false-positive case of IONM. Therefore, we suggest that the specificity and NPV should be considered as more objective indicators when interpreting the diagnostic efficacy of IONM. Taken together, given the nature of IONM, a low ratio of false negatives can reflect its accuracy, as well as favorable outcomes.

We found that consideration of any EP changes when interpreting the IONM resulted in the lowest negative LR value. Therefore, to ensure patient safety, rather than relying on only one modality, we recommend employing both EP modalities and interpreting the results accordingly (32, 33). The coverage differences between MEP and SSEP during IONM is indicative that both MEP and SSEP should be considered. MEP mainly reflects the functional integrity of the motor pathway (34). On the other hand, SSEP reflects the overall cerebral cortical perfusion as well as the functional integrity of the sensory pathway (35). Therefore, MEP and SSEP respond better to subcortical and cortical ischemia, respectively, depending on the anatomical differences and characteristics of the pathways of both EPs (36). Additionally, given the location of the motor pathway, a lower extremity SSEP may be more sensitive to ischemia than MEP during anterior choroidal artery or ACA territory UIA clipping (19, 37). Case 261 involved PC application for repairing an Acom UIA, where a decrease in lower extremity SSEP amplitude was observed as an ACA infarction. However, the amplitude in MEP monitoring was maintained within the acceptable range throughout the operation. Therefore, even without MEP satisfying the warning criteria, IC can occur with abnormal SSEP findings only. Particularly, in the non-MCA territory, given the high likelihood of a false-negative MEP, care should be taken when interpreting the monitoring results (38, 39). Although both the MEP and SSEP pathways are mainly distributed in the MCA territory, our risk factor analysis showed that UIA in the non-MCA territory was not a significant risk factor for IC.

Irreversible EP change showed very high specificity and NPV. PND occurred in 88.9% of patients with irreversible EP change. This finding, similar to those of previous studies, reports an association of persistent EP changes with poor outcomes (12, 40). Therefore, in the case EP changes do not intraoperatively recover, aggressive rescue interventions through rapid cause detection and correction should be performed. Moreover, immediate evaluations and aggressive therapeutic interventions should be performed immediately after surgery. Given that all four patients with transient PND showed irreversible EP changes, EP irreversibility might not necessarily be associated with symptom severity. On the other hand, among 18 patients with reversible EP change, only one showed PND. We presumed that the EP reversibility reflected the correction or stopping of the neural insult to a minimal level through intraoperative rescue interventions. Therefore, the presentation of EP changes during IONM requires immediate and active corrections, which is also critical for patients' long-term prognosis (41).

Our study has several limitations. Firstly, this was a single-center retrospective study. Secondly, the recruitment periods for the M and N groups differed; therefore, selection bias might be present. Thirdly, since the overall study period was 8 years, there could have been differences in the operative procedures and surgeons within the study period. Fourthly, we performed functional assessments based on the mRS score, which cannot reflect a detailed neurologic state. Specifically, non-motor complications, including cognitive, language, and swallowing problems, have been masked. Finally, there was a relatively small number of cases showing positive outcomes, which could have affected the statistical power. A large-scale, multicenter study based on standardized IONM protocol and detailed clinical data needs to be carried out in the future.

## Conclusions

IONM during UIA clipping significantly lowers the risks of sustained PND, IC, and radiologic complications. Surgeons can widen the scope of their surgical decisions using IONM. Moreover, maximum patient safety can be guaranteed through appropriate rescue interventions for EP changes caused by intraoperative events. The diagnostic effectiveness of IONM was optimal when changes in any EP modality were considered. In particular, IONM specificity and NPV were high. Consequently, IONM is a useful and essential tool for UIA clipping surgery.

## Data Availability Statement

The raw data supporting the conclusions of this article will be made available by the authors, without undue reservation.

## Ethics Statement

The studies involving human participants were reviewed and approved by The Institutional Review Board of Pohang Stroke and Spine Hospital. Written informed consent for participation was not required for this study in accordance with the national legislation and the institutional requirements. Written informed consent was obtained from the individual(s) for the publication of any potentially identifiable images or data included in this article.

## Author Contributions

DP contributed to the conception, design of the work, data collection, interpretation, and writing the manuscript. BK, S-EL, EJ, JP, and KC helped with the validation, investigation, and data analysis. Y-JC, SJ, and DH helped with the diagnosis and procedures of patients. M-CK edited the manuscript and supervised the entire study. All authors reviewed the manuscript and were in full agreement to submission.

## Conflict of Interest

The authors declare that the research was conducted in the absence of any commercial or financial relationships that could be construed as a potential conflict of interest.
