# Supplementary material for: Usefulness of Intraoperative Neurophysiological Monitoring During the Clipping of Unruptured Intracranial Aneurysm: Diagnostic Efficacy and Detailed Protocol
Source: Front Surg. 2021 Feb 26;8:631053. doi: 10.3389/fsurg.2021.631053 (PMC7952634; doi:10.3389/fsurg.2021.631053)
Supplement: Supplementary file 1 [file Table_1.DOCX]

**Supplementary Table 1. The distribution of aneurysm locations in cases of PND and IC**

|  | PND | | |  | IC | | |
| --- | --- | --- | --- | --- | --- | --- | --- |
|  | M group^a^ | N group | *p* value |  | M group^a^ | N group | *p* value |
| Vessel, n (%) |  |  | 0.52 |  |  |  | 0.26 |
| MCAB | 6 (54.5) | 3 (23.1) |  |  | 6 (75.0) | 3 (30.0) |  |
| MCA | 1 (9.1) | 4 (30.8) |  |  | 0 (0.0) | 3 (30.0) |  |
| Acom | 2 (18.2) | 3 (23.1) |  |  | 1 (12.5) | 2 (20.0) |  |
| ACA | 0 (0.0) | 0 (0.0) |  |  | 0 (0.0) | 0 (0.0) |  |
| Pcom | 1 (9.1) | 1 (7.7) |  |  | 1 (12.5) | 1 (10.0) |  |
| Acho | 0 (0.0) | 2 (15.4) |  |  | 0 (0.0) | 1 (10.0) |  |
| ICA | 1 (9.1) | 0 (0.0) |  |  | 0 (0.0) | 0 (0.0) |  |
| Non-MCA | 4 (36.4) | 6 (46.2) | 0.63 |  | 2 (25.0) | 4 (40.0) | 0.50 |
| MCA | 7 (63.6) | 7 (53.8) |  |  | 6 (75.0) | 6 (60.0) |  |

PND, postoperative neurologic deficit; IC, ischemic complications; MCAB, middle cerebral artery bifurcation; MCA, middle cerebral artery; Acom, anterior communicating artery; ACA, anterior cerebral artery; Pcom, posterior communicating artery; Acho, anterior choroidal artery; ICA, internal carotid artery.

^a^one case of multiple aneurysms

**Supplementary Table 2. Patients according to each EP change pattern and PND**

| Applied modalities |  | PND (tra/sus) | Normal |
| --- | --- | --- | --- |
| Changes in any EP  (MEP or/and SSEP) | Deteriorated | 9 (4/5) | 19 |
|  | No change | 1 (0/1) | 290 |
|  |  |  |  |
| Changes in all EP  (both MEP and SSEP) | Deteriorated | 2 (2/0) | 3 |
|  | No change | 8 (2/6) | 306 |
|  |  |  |  |
| Changes in MEP | Deteriorated | 5 (3/2) | 7 |
|  | No change | 5 (1/4) | 302 |
|  |  |  |  |
| Changes in SSEP | Deteriorated | 6 (3/3) | 15 |
|  | No change | 4 (1/3) | 294 |
|  |  |  |  |
| Reversible EP changes | Deteriorated | 1 (0/1) | 18 |
|  | No change | 9 (4/5) | 291 |
|  |  |  |  |
| Irreversible EP changes | Deteriorated | 8 (4/4) | 1 |
|  | No change | 2 (0/2) | 308 |

EP, evoked potentials; PND, postoperative neurologic deficit; tra,transient; sus,sustained; MEP, motor evoked potential; SSEP, somatosensory evoked potential.

**Supplementary Table 3. Features of temporary clipping cases**

|  | M group | N group | *p* value |
| --- | --- | --- | --- |
| Patients, n | 26 | 13 |  |
| IC with TC, n (%) | 2 (7.7) | 3 (23.1) | 0.31 |
| Aneurysm, n | 29 | 13 |  |
| Duration, minutes (range) | 5 (1–10) | 5 (4–7) | 0.99 |
| Reason of TC application |  |  | 0.17 |
| Premature bleeding | 7 (24.1) | 6 (46.2) |  |
| Aneurysm remodeling | 22 (75.9) | 7 (53.9) |  |
| Vessel, n (%) |  |  | 0.91 |
| MCAB | 16 (55.2) | 7 (53.8) |  |
| MCA | 3 (10.3) | 2 (15.4) |  |
| Acom | 4 (13.8) | 1 (7.7) |  |
| ACA | 1 (3.4) | 1 (7.7) |  |
| Pcom | 3 (10.3) | 1 (7.7) |  |
| Acho | 2 (6.9) | 0 (0.0) |  |
| ICA | 0 (0.0) | 1 (7.7) |  |

IC, ischemic complications; TC, temporary clinpping; MCAB, middle cerebral artery bifurcation; MCA, middle cerebral artery; Acom, anterior communicating artery; ACA, anterior cerebral artery; Pcom, posterior communicating artery; Acho, anterior choroidal artery; ICA, internal carotid artery.
